# Exosome: The “Off-the-Shelf” Cellular Nanocomponent as a Potential Pathogenic Agent, a Disease Biomarker, and Neurotherapeutics

**DOI:** 10.3389/fphar.2022.878058

**Published:** 2022-05-24

**Authors:** Satyajit Ghosh, Surajit Ghosh

**Affiliations:** Department of Bioscience and Bioengineering, Indian Institute of Technology Jodhpur, Karwar, India

**Keywords:** exosome, disease biomarker, cell-cell communication, neurotherapeutics, exosome engineering, pathogenic agent

## Abstract

Exosomes are nanosized “off-the-shelf” lipid vesicles released by almost all cell types and play a significant role in cell–cell communication. Exosomes have already been proven to carry cell-specific cargos of proteins, lipids, miRNA, and noncoding RNA (ribonucleic acid). These vesicles can be selectively taken up by the neighboring cell and can regulate cellular functions. Herein, we have discussed three different roles of exosomes in neuroscience. First, we have discussed how exosomes play the role of a pathogenic agent as a part of cell–cell communication and transmit pathogens such as amyloid-beta (Aβ), further helping in the propagation of neurodegenerative and other neurological diseases. In the next section, the review talks about the role of exosomes in biomarker discovery in neurological disorders. Toward the end, we have reviewed how exosomes can be harnessed and engineered for therapeutic purposes in different brain diseases. This review is based on the current knowledge generated in this field and our comprehension of this domain.

## 1 Introduction

**FIGURE 1:**
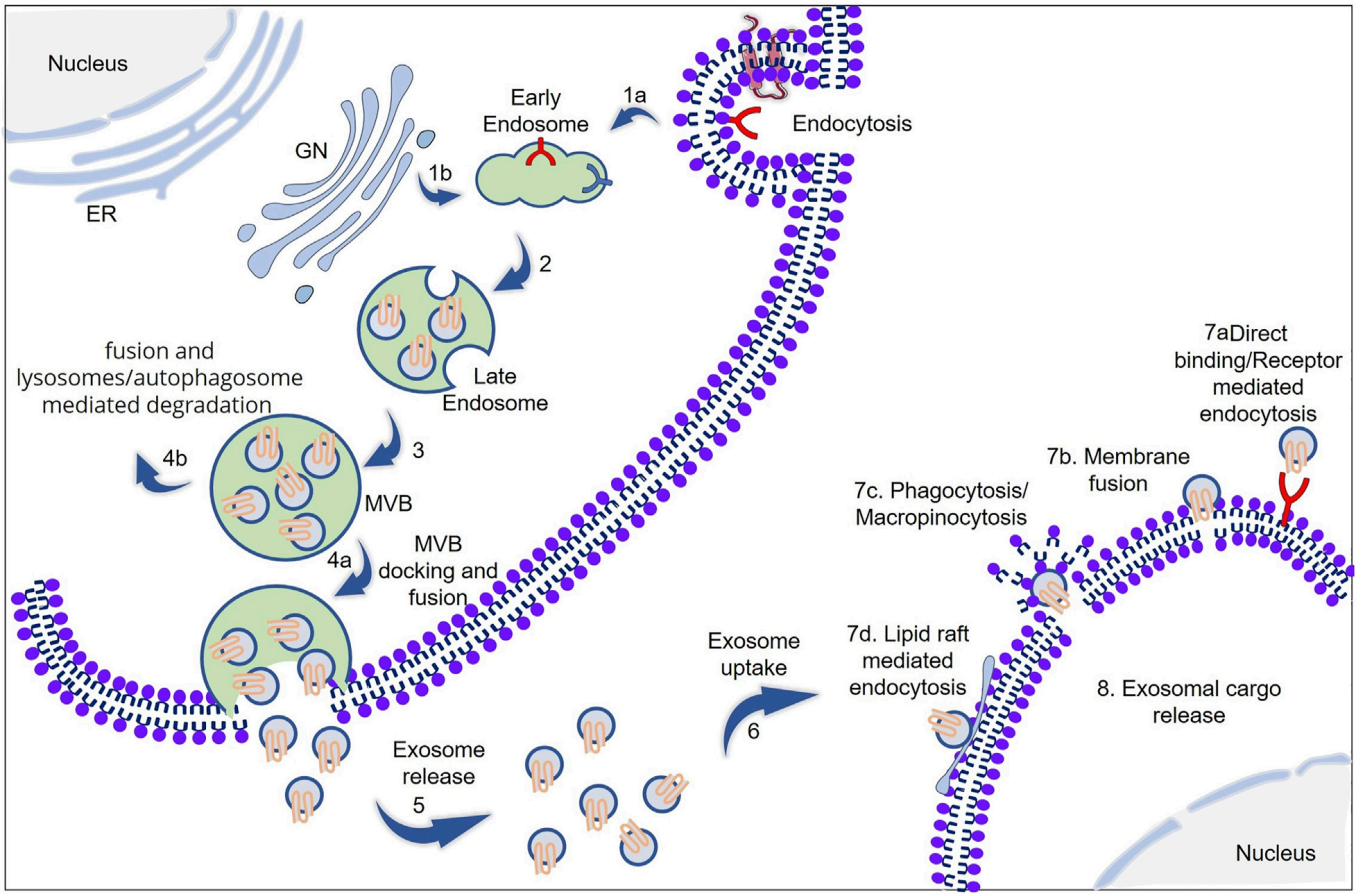
Schematic representation of exosome biogenesis, cargo packing, and cellular uptake. **(1a,b)** Early endosomes are formed from the inward budding of the plasma membrane or Golgi network (GN). **(2,3)** Early endosomes mature to form late endosomes and subsequently to multivesicular bodies (MVB). **(4a)** MVB docking into the plasma membrane *via* SNAREs and SNAP23, and subsequent fusion with the plasma membrane results in exosome release **(5)**. Alternatively, MVBs can fuse with lysosome and subsequent degradation **(4b)**. **(6)** The released exosome will be taken up by the neighboring cell. The cellular uptake of exosomes can be **(7a)** direct binding and receptor-mediated endocytosis, **(7b)** membrane fusion, **(7c)** phagocytosis/micropinocytosis, or **(7d)** lipid raft-mediated endocytosis. The ESCRT machinery plays a key role in protein sorting, particularly for ubiquitinated cargos. During the process of exosome biogenesis, various proteins, including RNA-binding proteins (RBPs), are selectively sequestered into exosomes; these RBPs help in RNA cargo packing into the exosome. (ER: endoplasmic reticulum; GN: Golgi network; MVB: multivesicular bodies; ESCRT: endosomal sorting complex required for transport).

Exosomes, saucer-shaped vesicles of approximately 30–100 nm diameter (Théry et al., 2002b), are one of the different types of “extracellular vesicle” (EV) that are delimited by a lipid bilayer and are naturally released from the cell and play major roles in cell–cell communications. These vesicles are endosomal origin, cannot replicate, i.e., do not contain a functional nucleus, and float at a density of 1.13–1.19 g ml^−1^ in sucrose gradients (Théry et al., 2018). The process of exosome release can be divided into three steps: exosome biogenesis, intracellular movement of multivesicular bodies (MVBs), and MVB fusion with the plasma membrane. In the first step of exosome biogenesis (Figure 1), early endosomes are formed by inward invagination of the plasma membrane or from the trans-Golgi network. These early endosomes mature to form late endosomes. Invagination of the endosomal membrane into the lumen leads to the formation of intraluminal vesicles (ILVs), which finally leads to the generation of MVBs. Lastly, the generated MVBs will fuse with the plasma membrane or alternatively with lysosomes or autophagosomes. The former results in the release of the exosome, and the latter results in the degradation of MVBs. Several molecules are involved in this complicated process, and the details are summarized elsewhere (Teng and Fussenegger, 2021).

**FIGURE 2:**
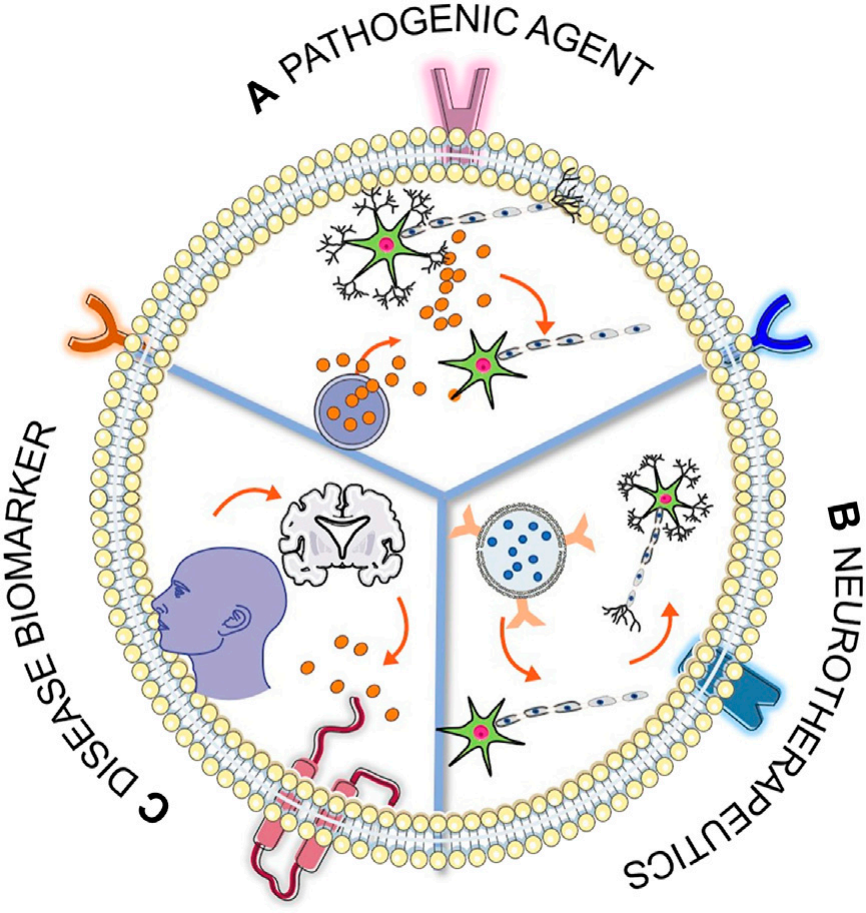
Schematic representation of roles of exosomes in three different fields. **(A)** The blue sphere represents MVB, and the red sphere represents the exosome; this section of the figure represents the release of pathogenic cargo from the exosome and subsequent disease transmission. **(B)** Therapeutic potential of surface engineered cargo loaded exosome and subsequent recovery from disease condition. **(C)** Exosomes released from the disease-affected cell can cross the BBB and can be found in blood circulation, which can be utilized in noninvasive biomarker discovery. [Some component of the figure is adapted from Servier Medical Art; Servier is licensed under a creative commons attribution 3.0 unported license (https://smart.servier.com/)].

Exosomes are not merely lipid vesicle; they also contain membrane-associated proteins, transmembrane proteins, mRNA, noncoding RNAs, and other cell-specific cargos. Exosomes are equipped with endosomal sorting complex required for transport (ESCRT), Alix, TSG101, HSC70, CD63, CD81, and HSP90β protein that help in the formation and release of exosomes. CD63 and CD81 (Theos et al., 2006; Stuffers et al., 2009) are tetraspanin family proteins that are thought to help in exosome formation and release by an ESCRT-independent mechanism. These proteins are enriched in exosomes compared to the cell lysate, and these are termed “exosomal marker proteins,” which can be identified using Western blot with appropriate antibodies (Thompson et al., 2016; Doyle and Wang, 2019). ExoCarta database hosts about 41,860 proteins, >7540 RNA, and 1,116 lipid molecules from more than 286 exosomal studies; this can help us to have an idea about the diversity of exosomal content from different cell types, current cell state (e.g., transformed, differentiated, stimulated, and stressed), and culture conditions (Keerthikumar et al., 2016). A different method of isolation of exosomes from condition media has been introduced, from the most commonly used methods ultra-centrifugation, sucrose gradient centrifugation, different kit-based to microfluidics-based methods (Supplementary Table S1) (Théry et al., 2006; Wu et al., 2017). The choice of exosome isolation method greatly impacts the exosome quality and quantity (Patel et al., 2019; Brennan et al., 2020). The isolated exosomes can be characterized using nanoparticle tracking analysis (NTA) for size distribution and surface charge, transmission electron microscopy for size distribution and morphology (TEM), atomic force microscopy (AFM) for size distribution and surface morphology, and Western blotting to check the presence and absence of protein markers (Wu et al., 2015; Chopra et al., 2019). Experimental evidence and live-cell imaging (Sung et al., 2020) have already shown that exosomes are secreted by all cell types (Peters et al., 1989; Raposo et al., 1996; Théry et al., 2002a; Morelli et al., 2004) and brain cells like neurons, astrocytes, microglia, and oligodendrocytes are not the exception (Potolicchio et al., 2005; Fauré et al., 2006; Krämer-Albers et al., 2007; Taylor et al., 2007). In the late 1980s, when exosome was first discovered, it was thought to be cellular waste resulting from cell damage or by-products of cell homeostasis and had no significant impact on neighboring cells (Johnstone et al., 1987). But currently, it is crystal clear that exosomes and their cargo can play a major role in cellular processes like in immune response (Greening et al., 2015), signal transduction (Gangoda et al., 2015), antigen presentation (Mittelbrunn et al., 2011) as well as in disease state like chronic inflammation (Lässer et al., 2016), cardiovascular and renal diseases (Gonzalez-Calero et al., 2014), neurodegenerative diseases (Howitt and Hill, 2016), lipid metabolic diseases (Record et al., 2014), traumatic brain injury (Zhang et al., 2021), mental disorder (Saeedi et al., 2019), and tumors (Salem et al., 2016). It is now a well-established fact that exosomes can be found in almost all biological fluids like blood (Hornung et al., 2020), urine (Street et al., 2017), saliva (Machida et al., 2015), breast milk (Qin et al., 2016), cerebrospinal fluid (Yagi et al., 2017), semen (Madison et al., 2015), and amniotic fluid (Keller et al., 2007). Exosomes isolated from these fluids will reflect the cellular origin and its physiological state as a “fingerprint” or “signature” of the donor cell. From this, it is very clear that exosomes can be a potential target for biomarker discovery and early detection of many diseases. Apart from the role of exosomes in signal transduction and biomarker discovery, they can also be harnessed to be used as therapeutics in many diseases (Cooper et al., 2014; Zhuang et al., 2011). The ability to cross blood–brain barrier (BBB), non-immunogenicity, the option of surface engineering, and selective cargo packaging make exosomes emerge as a blockbuster therapeutic option in many diseases (Ghosh et al., 2020; Zhan et al., 2020; Mishra et al., 2021). The probable roles that can be played by exosomes are schematically summarized in Figure 2.

## 2 Exosome as a Pathogenic Agent in Neurological Diseases

**FIGURE 3:**
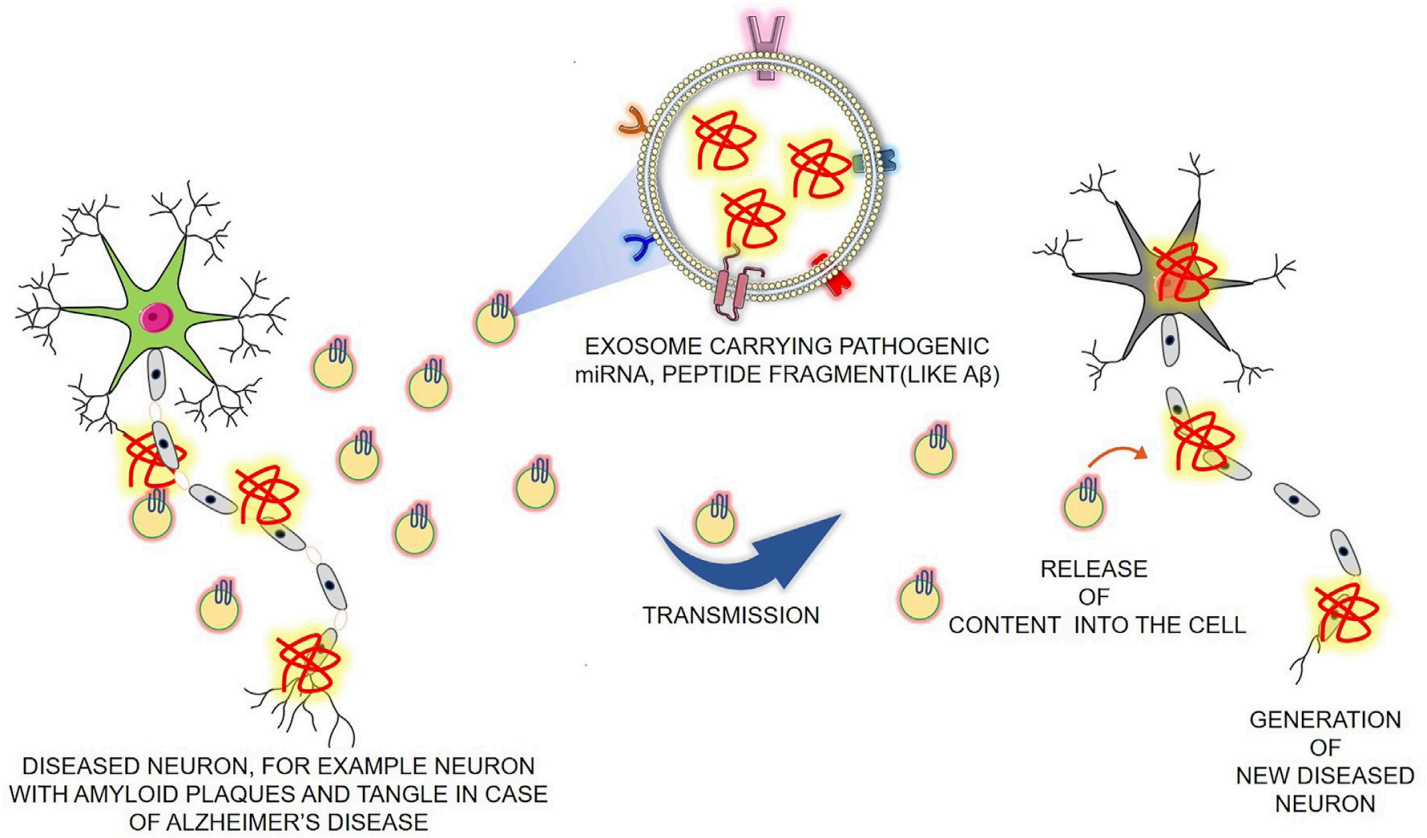
Schematic representation of the role of exosomes as the pathogenic carrier in neurodegenerative disease. Pathogenic cargo-loaded exosomes are released from the diseased cell, and the cells that take up the exosomes get a similar kind of disease. This picture represents a similar phenomenon, taking an example of how exosomes can carry Aβ pathogenic peptides in their lumen and cause the propagation of Alzheimer’s disease. (Aβ: amyloid-beta) (Some component of the figure is adapted from Servier Medical Art; Servier is licensed under a creative commons attribution 3.0 unported license (https://smart.servier.com/).

The cells in the central nervous system (CNS) communicate between themselves by intercellular and extracellular interactions. The former is mediated by ions and can be transduced and sensed by the cell through ion channels and neurotransmitter receptors present in neurons and glial cells (Yamazaki et al., 2007; Debanne and Rama, 2011). The latter could consist of either wiring transmission, which is primarily dependent on synapses or volume transmission, mediated by exosome for major vesicular carrier or by exocytosis of neurotransmitters (Trueta and De-Miguel, 2012; Borroto-Escuela et al., 2015). The secretion of exosomes from CNS cells was first demonstrated in cultured embryonic cortical neurons, and it can be released either presynaptically at the neuromuscular junction or postsynaptically by cortical neurons upon activation of synaptic NMDA receptors, which will then bind presynaptically to hippocampal neurons (Fauré et al., 2006; Zhang and Yang, 2018). Experimental evidence has also shown that neuron-derived exosomes are 50 times more abundant in soma and dendrites than axons in both peripheral nervous system (PNS) and CNS (Von Bartheld and Altick, 2011). In the brain, the exosomes act as local or distant messengers and communicators and can play a significant role in neural homeostasis, modulation of synaptic plasticity, synaptic transduction, modifying the cell surface properties of target cells, auto-protective mechanism for neurons, sequestering “toxic” (pathogenic) proteins, and promoting regeneration and neuroprotection both in the CNS and the PNS (Korkut et al., 2013; Lopez-Verrilli et al., 2013; Chivet et al., 2014; Kalani et al., 2014; Hornung et al., 2020). In addition to interneuronal communication, exosomes from neuronal culture when added to astrocyte culture have shown to have an effect in extracellular glutamate levels and modulation of synaptic activation (Morel et al., 2013). In the opposite case, when glial cell-derived exosomes are added to the neuronal culture, they significantly increase the firing rate of neurons and has a neuroprotective role under oxidative stress and starvation conditions (Smalheiser, 2007; Morel et al., 2013; Fröhlich et al., 2014). Apart from normal brain function, it is already a proven fact that exosomes have a role in disease progression and can act as a pathogen delivery agent (Hornung et al., 2020; Zhang et al., 2021). The different roles of exosomes in disease are as follows and are schematically represented in Figure 3.

### 2.1 Alzheimer’s Disease

Extracellular deposition of polymerized amyloid-β (Aβ) protein, also called plaques, and intracellular filamentous inclusions of hyperphosphorylated tau protein, known as neurofibrillary tangles, are the two major neuropathology involved in Alzheimer’s disease (AD) (Yamaguchi et al., 1989; Lee et al., 1991). Several lines of evidence pointed toward the fact that the exosomes have a role in amyloid pathology in AD. Scientists have shown that in HeLa and N2a cells, after beta-cleavage of amyloid precursor protein (APP) in early endosomes, a minute fraction of Aβ peptides can be secreted from the cells in association with exosomes; not only that but also exosomal proteins have also been found to accumulate in the plaques of AD patients’ brains (Rajendran et al., 2006). Intraperitoneal injection of GW4869, the moiety responsible for inhibition of neutral sphingomyelinase 2 (nSMase2) in the 5XFAD mouse, shows reduced levels of brain and serum exosomes, brain ceramide, and also reduces Aβ1–42 plaque load. This result suggests that exosomes are involved in the generation of Aβ plaques (Dinkins et al., 2014). In recent years, it has been experimentally proven that exosomes isolated from AD brains contain elevated levels of amyloid-beta oligomers; these exosomes can act as vehicles for the neuron-to-neuron transfer of such toxic species. Inhibition of the formation, secretion, or uptake of exosomes has been found to reduce both the spread of oligomers and the related toxicity (Sinha et al., 2018). Recent experimental evidence indicates that soluble pre-fibrillar Aβ species are more toxic than insoluble fibrils (Ladiwala et al., 2012). Scientists have shown that the exosomes of microglial origin are strikingly high in AD patients and in subjects with mild cognitive impairment and are toxic for cultured neurons. Studies have also found that the neurotoxicity of these exosomes is due to the capability of exosome lipids to promote the formation of soluble Aβ species and from the trafficking of neurotoxic Aβ *via* exosomes after Aβ got internalized into microglia (Joshi et al., 2014). Apart from Aβ, exosomes are also involved in tauopathy. A recent study shows much of the tau phosphorylated at Thr-181 is secreted by M1C cells and occurs *via* exosomal release (Saman et al., 2012). A group of scientists developed an adeno-associated virus-based model exhibiting rapid tau propagation from the entorhinal cortex to the dentate gyrus and has shown that microglia spread tau *via* exosomes secretion. Inhibiting exosome synthesis significantly reduces tau propagation *in-vitro* and *in-vivo* (Asai et al., 2015). Another group of scientists has discovered cambinol, an inhibitor of the neutral sphingomyelinase 2 (nSMase2) enzyme, and shown that cambinol works in a dose-dependent manner and suppresses extracellular vesicle (EV) production, which in turn reduce tau seed propagation (Bilousova et al., 2018). Apolipoprotein E (apoE) and bridging integrator-1(Bin1), the genetic risk factors for late-onset AD (LOAD), are involved in exosome biogenesis and cargo sorting (Cohn et al., 2021). Experiments have shown that overexpression of BIN1 in PS19 mice promotes the release of Tau *via* extracellular vesicles (Crotti et al., 2019). On the other hand, the apolipoprotein E4 genotype is involved in the downregulation of exosome biosynthesis and release; this will lead to decreased elimination of materials from the endo-lysosomal system. The failure of the endo-lysosomal system will contribute to amyloidogenic amyloid-β precursor protein processing, compromise trophic signaling and synaptic function, and interfere with a neuron’s ability to degrade material, all of which will result in neuronal vulnerability and a higher risk of AD development (Peng et al., 2019).

### 2.2 Parkinson's Disease

Parkinson’s disease (PD) is the second most common neurodegenerative disease (Lebouvier et al., 2009), which is characterized by the degenerative death of dopaminergic (DA) neurons in the substantia nigra, a significant decrease in striatal DA content, and the appearance of Lewy bodies due to the accumulation and aggregation of α-synuclein (α-syn) in the cytoplasm of residual nigrostriatal neurons (Spillantini et al., 1998). Experimental evidence has shown that α-syn is packaged into exosomes *via* the endosome pathway, and it can fuse with the plasma membrane for secretion as exosomal cargo with the assistance of VPS4 and SUMO proteins (Cabin et al., 2002; Lee et al., 2005). Recent studies suggest that exosomes provide favorable conditions for α-syn aggregate formation; this, in turn, promotes the propagation of PD pathology (Grey et al., 2015). Scientists have already discovered the presence of oligomeric α-syn in the exosome, which is readily taken up by the neighboring cell and is more toxic as compared to free α-syn (Danzer et al., 2012). In an experiment with an SH-SY5Y cell line, overexpressing α-syn has shown to have α-syn in isolated exosomes, and this can get transferred to normal SH-SY5Y cells (Alvarez-Erviti et al., 2011a). Later, it was also shown that the exosome-packed α-syn could promote the cell death of recipient neuronal cells. These experiments provide support to the hypothesis of exosome-mediated α-syn propagation between neurons and facilitate PD progression (Emmanouilidou et al., 2010). Recent research by a group of scientists suggested that the presence of α-syn oligomers in CD11b + exosomes of microglia origin can induce α-syn aggregation within neurons (Guo et al., 2020). Overall, it is very clear that exosomes can act as a pathogenic agent in PD propagation.

### 2.3 Frontotemporal Dementia and Amyotrophic Lateral Sclerosis

FTD involves progressive deficits in behavior, executive function, or language (Bang et al., 2015). Transactive response DNA-binding protein (TDP-43), its aggregation, and cytoplasmic translocation are thought to represent significant steps in the pathogenesis of FTD or ALS (Hu and Grossman, 2009). ALS is a distinct neurodegenerative disease affecting motor neurons in the brain and spinal cord; SOD1 was the first gene discovered to cause familial ALS and was the most studied cause of ALS (Sheng et al., 2012). FTD and ALS appear to be on a spectrum, and some patients display mixed phenotypes of both diseases (Kawakami et al., 2019). The TDP-43 gene is involved in the pathogenesis of both the disease, but SOD1 is only related to ALS but not FTD (Hornung et al., 2020; Jo et al., 2020). Using mouse motor neuron-like NSC-34 cells overexpressing human wild-type or mutant SOD1, scientists have shown that exosomes derived from NSC-34 cells contain SOD1; this gave the evidence of secretion and cell-to-cell transmission of SOD1 (Gomes et al., 2007). In the similar way, TDP-43 can also get transmitted from cell to cell (Nonaka et al., 2013; Iguchi et al., 2016).

### 2.4 Traumatic Brain Injury

TBI occurs due to the sudden external force in the brain that leads to temporary or permanent neurological deficits. TBI pathogenesis is a complex process due to primary and secondary injuries. The primary deficit occurs immediately, and the secondary injury can occur from minutes to days after the primary impact and consists of a molecular, chemical, and inflammatory cascade responsible for further cerebral damage. The injury involves depolarization of the neurons and release of excitatory neurotransmitters such as glutamate and aspartate that lead to increased intracellular calcium levels, which in turn activates caspases and free radicals that result in the degradation of cells either directly or indirectly through an apoptotic process. These cell deaths result in an inflammatory response that further damages neuronal cells and the blood–brain barrier (BBB) and promotes cerebral edema. The secondary injury phase is followed by the recovery period that involves reorganization at an anatomical, molecular, and functional level. The brain parenchyma, cerebrospinal fluid, and blood make up the volume of the intracranial compartment. An increase in intracranial volume *via* mass effect from blood, both cytotoxic and vasogenic edema, and venous congestion is also a hallmark of TBI. This would lead to pathological brain compression and, finally, death (Galgano et al., 2017). Exosomes are actively participating in traumatic brain injury pathogenesis; in a case study involving military personnel with mild TBIs (mTBI) and chronic symptoms, it is found that there is a higher level of tau, amyloid-beta 42, and IL-10 in neuron-derived exosomes (NDEs) (Gill et al., 2018). A group of scientists postulated that exosomes could mediate the induction of chronic traumatic encephalopathy (CTE) from mTBI. They have hypothesized a pathway to show how exosomes can mediate pathogenesis from normal to mild deterioration after one mTBI to advanced CTE pathology after the repeated occurrence of mTBIs. According to their hypothesis, initial mTBI leads to the production of NDEs that contains pathogenic complexes of PRPc-Abo-Fyn, SNGY3 + P-tau, and IL-6-sIL-6R; this leads to damage in the donor neurons and other neurons that receive the neurotoxic NDEs. Apart from the neurons, microglia (MG) and astrocytes (AG) will also produce microglia-derived exosomes (MDEs) and astrocytes-derived exosomes (ADEs), respectively, carrying elevated levels of APP, BACE-1, and IL-6, which will further cause neurotoxic damage to neurons. With subsequent episodes of mTBI, this series of processes will increase and cause neuronal apoptosis, which will subsequently lead to the induction of CTE (Goetzl et al., 2019). From these studies, it is believable that the NDE from TBI patients contains neurotoxic cargo, and this NDE causes neuronal damage in proximal or distal cells that receive it.

### 2.5 Glioblastoma

Apart from its role as a mediator of neurodegenerative disease, exosomes also play a significant role as a pathogenic agent in brain malignancies. The most frequent intrinsic tumors of the central nervous system are glioma. It encompasses two principal subgroups: (World Health Organisation) WHO grade I or “nondiffuse gliomas,” showing a more circumscribed growth pattern, and WHO grades II–IV or “diffusely infiltrating gliomas,” arising from glial cells or glial precursors (Wesseling and Capper, 2018). Scientifically accepted hallmark of cancers includes sustaining proliferative signaling, resisting cell death, evading growth suppression, activating invasion and metastasis, enabling replicative immortality, and inducing angiogenesis (Hanahan and Weinberg, 2011). Exosomes play a significant role as a pathogenic agent in all the six hallmark scenarios (Choi et al., 2018; Oushy et al., 2018; Bian et al., 2019; Hallal et al., 2019; Gao et al., 2020; Lucero et al., 2020). Apart from these roles, studies have shown that glioblastoma-derived exosomes can promote the immunosuppressive properties of microglia when they are taken up by tumor-associated microglia (Abels et al., 2019). Recent studies have shown that exosomes have active participation in the acquisition of resistance to therapy in glioblastomas (Yekula et al., 2021). From the aforementioned evidence, it is clear that exosomes play a major role as a pathogenic agent in maintaining the tumor microenvironment and further metastasis of tumors.

## 3 Exosome in Biomarker Discovery for Neurological Diseases

**FIGURE 4:**
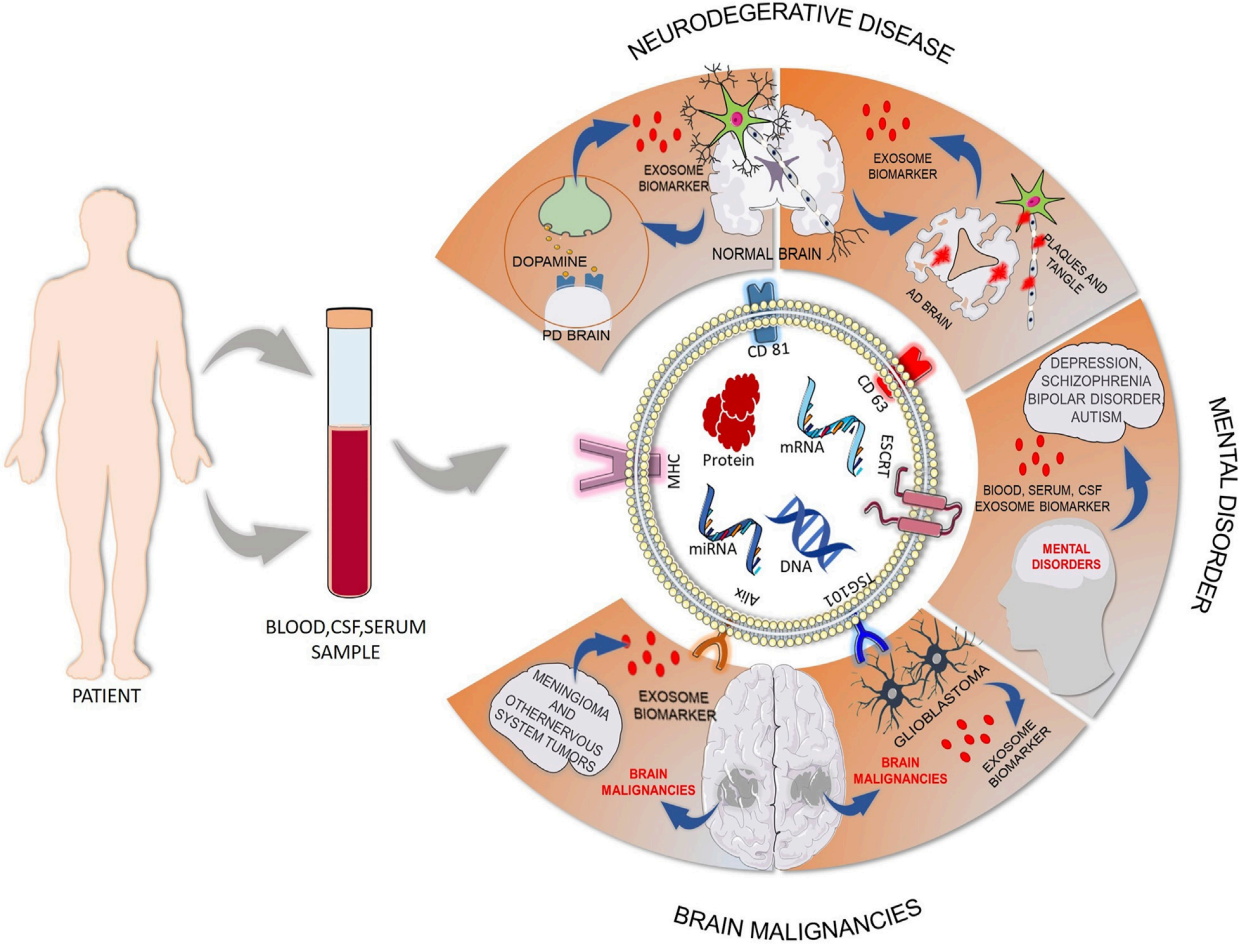
Exosome-based biomarker discovery in neurological disorders from biofluids. Blood, cerebrospinal fluid, plasma, or serum sample can be collected from patients, and the content of the isolated exosomes from that sample can give us the opportunity in early detection of disease to gain knowledge about disease state and disease severity. This figure gives a snapshot of exosome release from disease states like neurogenerative disease, mental disorder, and brain malignancies and the biomarker potential of exosomes. (AD: Alzheimer’s disease; PD: Parkinson's disease) [Some component of the figure is adapted from Servier Medical Art; Servier is licensed under a creative commons attribution 3.0 unported license (https://smart.servier.com/)].

The blood–brain barrier (BBB) is a complex physical barrier between the brain and the peripheral circulation that regulates the influx and efflux of molecules to the brain to preserve CNS homeostasis and maintains the stable local ionic microenvironment necessary for neuronal function (Armulik et al., 2010; Kheirandish-Gozal et al., 2017). This barrier makes it difficult for biomolecules to pass from the brain side to the peripheral circulation and remains the main obstacle in the discovery of biomarkers from peripheral blood or serum for brain-related diseases. After the discovery that exosomes can cross BBB and the exosomal content remains active, the interest in exosome-based biomarker discovery in neurological disorder has increased (Saeedi et al., 2019). Exosomal content can help us gain insights into early disease detection, disease state, and disease severity (Figure 4). The ability to compare the biomarkers in exosomes originating from different cell types gives an added advantage to biomarker analysis in CNS-derived blood exosomes as compared to CSF (Hornung et al., 2020). Different biofluids and their exosomal content for biomarker discovery in neurological diseases are listed in Supplementary Table S2.

## 4 Exosome as Neurotherapeutics

In the previous section, we briefly introduced the role of exosomes as a biomarker and pathogenic agent. In this section, we will be focusing on the recent progress surrounding exosomal surface engineering and engineering exosome for packaging cargo of interest. We will also discuss how exosome engineering can increase the value of exosomes as therapeutics in different neurological disorders.

### 4.1 Engineered Exosome

The main issue with exosome therapeutics is it does not have targeting ability. There are two types of exosome engineering: surface engineering and packaging of cargo of interest. The former endows the exosomes with targetability, and the latter makes the exosomes a better delivery agent and increases therapeutic value.

#### 4.1.1 Methods of Exosome Engineering

Methods of exosome engineering involve two strategies: i) surface engineering strategy (Richardson and Ejima, 2019) and ii) exosome packaging strategy (Donoso-Quezada et al., 2020).

##### 4.1.1.1 Surface Engineering Strategy

Currently available methods for exosome surface functionalization can be classified into two main approaches: 1) genetic engineering and 2) chemical modification. The former method is effective for displaying genetically engineered proteins on the surface of exosomes, but it is only limited to genetically encodable peptides and proteins. But the latter chemical modification method can be used to functionalize exosomes with a wide range of molecules by utilizing noncovalent or covalent interactions. This method remains challenging because of the membrane complexity and because of the various issues with the purification steps necessary to separate the unreacted chemicals from the exosomes (Richardson and Ejima, 2019).

1. Genetic engineering-based surface engineering includes designing plasmids, transfecting cells with the designed plasmid, and exosomes isolation which itself is a challenging and expensive task. In many works, lactadherin that localizes to exosomes *via* binding of its C1C2 domain to exosome lipids has been utilized for the generation of chimeric protein and exosome functionalization (Delcayre et al., 2005). The protein of interest is cloned to the C1C2 domain of the lactadherin, which results in chimeric proteins being trafficked to the exosomes, and the N-terminal region was displayed outward on the exosome surface. Different scientific groups have utilized lactadherin to display GLuc reporter protein, carcinoembryonic antigen, and HER2 and anti-HER2 antibodies on the exosome surface (Hartman et al., 2011; Takahashi et al., 2013; Wang et al., 2018b). In another set, scientists have utilized exosomal membrane protein, Lamp2b, and fused targeting peptides, e.g., RVG and RGD peptides to the N terminus of the protein for exosome surface functionalization and targeted delivery to neurons (Kumar et al., 2007) and breast cancer cell (Tian et al., 2014). Yim et al. fused CIBN to the N-terminus of EGFP tagged CD9 (CIBN-EGFP-CD9) for the vector preparation and fused the cargo proteins with CRY2 (cargo protein-CRY2). This system helped to immobilize proteins to the inner surface of exosomes and loaded cargo proteins into the newly generated exosomes (Yim et al., 2016a). Lai et al. (2014) have generated a lentivirus vector encoding the transmembrane domain of PDGFR, BAP domain, and GLuc, and this construct generates surface-engineered exosomes with Gluc and BAP domain which gives an opportunity for *in vivo* multimodal imaging to monitor tissue distribution, blood levels, and clearance dynamics of the EV. Though the genetic engineering-based methods have advantages, this method still has risks of the engineered biomolecules appearing on the internal exosome surface rather than the desired external surface.
2. Chemical modification involves covalent or noncovalent interaction for exosome surface functionalization. Alkyl chains can be utilized to anchor molecules into the lipid bilayer membranes of exosomes through hydrophobic interactions. Using this strategy, PEG-lipid conjugates were inserted into exosomal membranes to increase blood circulation time (Kooijmans et al., 2016). Wan et al. (2018) utilized lipids to modify cell membranes for the formation of exosome-mimetic vesicles. They conjugated nucleolin-targeting aptamer AS1411 to cholesterol-PEG and generated surface-functionalized exosomes for *in vivo* anticancer drug delivery. Functionalized exosomes can also be generated by fusing liposomes, consisting of DOPS and PEG-DSPE with exosomes *via* freeze−thaw cycles. This method also justifies the efficient packing of liposomal cargo into the newly generated liposome fused exosome (Sato et al., 2016). Wan et al. (2017) have demonstrated the hybridization-mediated assembly of DNA on the exosomal surface for the generation of targeted exosomes. Apart from noncovalent modifications, exosomes can be covalently modified; these modifications are less prone to dissociate away from the exosomes, unlike noncovalent modifications. Smyth et al. (2014) modified the exosomes with alkyne-containing 4-pentynoic acid used carbodiimide coupling onto amines in the exosomal membrane. Then, they used these functionalized exosomes to conjugated azide-tagged fluorophores *via* azide−alkyne Huisgen cycloaddition or click chemistry. Wang et al. (2015) used metabolic engineering to introduce azide groups on the surface of exosomes; they have also used azide−alkyne Huisgen cycloaddition reaction to covalently introduce small molecules and proteins onto the exosomal surface. Many other works also used similar chemical-based approaches for surface engineering of exosomes (Qi et al., 2016; Kumar et al., 2018; Tian et al., 2018).

##### 4.1.1.2 Exosome Packaging Strategy

Several studies have explored the natural properties of exosomes as nanocarriers, and in recent years, numerous techniques have been developed to improve the immunogenicity, drug loading efficiency, or targeting ability of exosomes. In this section, we will discuss the state-of-the-art packaging strategies to load a cargo of interest into the exosome. Packaging strategies include

1. Passive loading of hydrophobic compounds: The lipidic nature of the exosome membrane enables several hydrophobic compounds to be passively loaded into the exosomes by co-incubation. Using this strategy, curcumin (Sun et al., 2010), doxorubicin, and paclitaxel (Yang et al., 2015) have been successfully incorporated into different cell-derived exosomes. Apart from therapeutic molecules, large protein, e.g., the tetrameric protein catalase, has efficiently been loaded into Raw 264.7-derived exosomes by simple diffusion (Haney et al., 2015).
2. Physical methods for molecule loading: The previously discussed passive loading method is not efficient for packaging hydrophobic molecules like DNA or RNA. In these scenarios, physical methods like electroporation, sonication, and extrusion-based methods are used. Momen-Heravi et al. (2014) loaded miRNA-155 into the exosomes using electroporation; they concluded that higher voltages (between 0.14 and 0.2 kV) and a total exosomal protein concentration between 500 and 1,000 μg/ml resulted in better loading yields. Wahlgren et al. (2012) used a similar method to load mitogen-activated protein kinase-1 siRNA (MAPK1-siRNA), and they have found that the optimum electroporation voltage was between 0.150 and 0.200 kV and the exosomal protein concentration was between 250 L and 1,000 μg/ml. Larger nucleic acids, like double-stranded DNA, have also been successfully packaged into exosomes by electroporation; it was found that the loading efficiency of dsDNA significantly decreases for sizes above 750 bp (Lamichhane et al., 2015). By using membrane-permeabilizing agents, the issue of aggregation and fusion of exosomes after electroporation can be resolved (Hood et al., 2014). Another physical method is sonication, and it is reported that sonication can successfully incorporate doxorubicin and paclitaxel into exosomes with more efficiency than other physical methods (Kim et al., 2016). Though this technique is more efficient as it is the most damaging technique for exosomal membrane, sonication is very rarely used for exosomal cargo packaging. Another less explored method for exosome cargo loading is cell extrusion, where cells are extruded through 100–400 nm pore size membrane filters to break up the cell and then cells reform the cell membrane to generate exosome-mimics. Using this method, exosome-mimics have been generated from MCF10A cells and loaded with siRNA by electroporation (Yang et al., 2016). Similarly, catalase was loaded into Raw 264.7-derived exosomes by extruding the catalase mixture with exosomes (Haney et al., 2015).
3. Hydrophobic modification of nucleic acids: To avoid the problem of aggregation, vesicle fusion, and variations of surface zeta potential associated with electroporation-based siRNA, miRNA cargo loading, hydrophobic modification of nucleic acids has evolved as a strategy to pack cargo into the exosomes. Didiot et al. have modified the siRNA by adding a cholesterol moiety conjugated to the 3’ end of the passenger strand and successfully loaded the modified cargo into U87 glioblastoma cell-derived exosomes (Didiot et al., 2016).
4. Labeling of target proteins for loading into exosomes: This method gives the opportunity to utilize the protein that plays a major role in exosomal cargo packaging; one such protein is ESCRT which specifically shortens the ubiquitinated proteins in the exosomes. In many studies, scientists are leveling cargo protein with a peptide that can selectively interact with ESCRT, which increases the probability of cargo protein getting packed into the exosomes (Villarroya-Beltri et al., 2014). Cheng and Schorey (2016) fused ubiquitin to the C-terminal region of enhanced green fluorescent protein (EGFP), tumor antigenic protein nHer2, and *Mycobacterium tuberculosis* proteins Ag58B and ESAT6; ubiquitin labeling increases the loading of all the protein into the exosomes. Other than ESCRT, late-domains (L-domains), which recognizes the WW tag in the protein of interest, also give similar opportunity in cargo loading (Sterzenbach et al., 2017).
5. Light-induced exosome loading: optically reversible protein–protein interaction (EXPLORE) can be used to load proteins into exosomes. This process involves endogenous biogenesis processes and the delivery of cargo proteins into the cytosol by light-mediated signal (Yim et al., 2016b). Scientists have explored exosomal CD9-CIBN-CRY2-based systems to pack many cargos into the exosomes (Yim et al., 2016a; Huang et al., 2019).

### 4.2 Applications of Cell-Derived and Surface-Engineered Novel Cargo-Loaded Exosome as Neurotherapeutics

**FIGURE 5:**
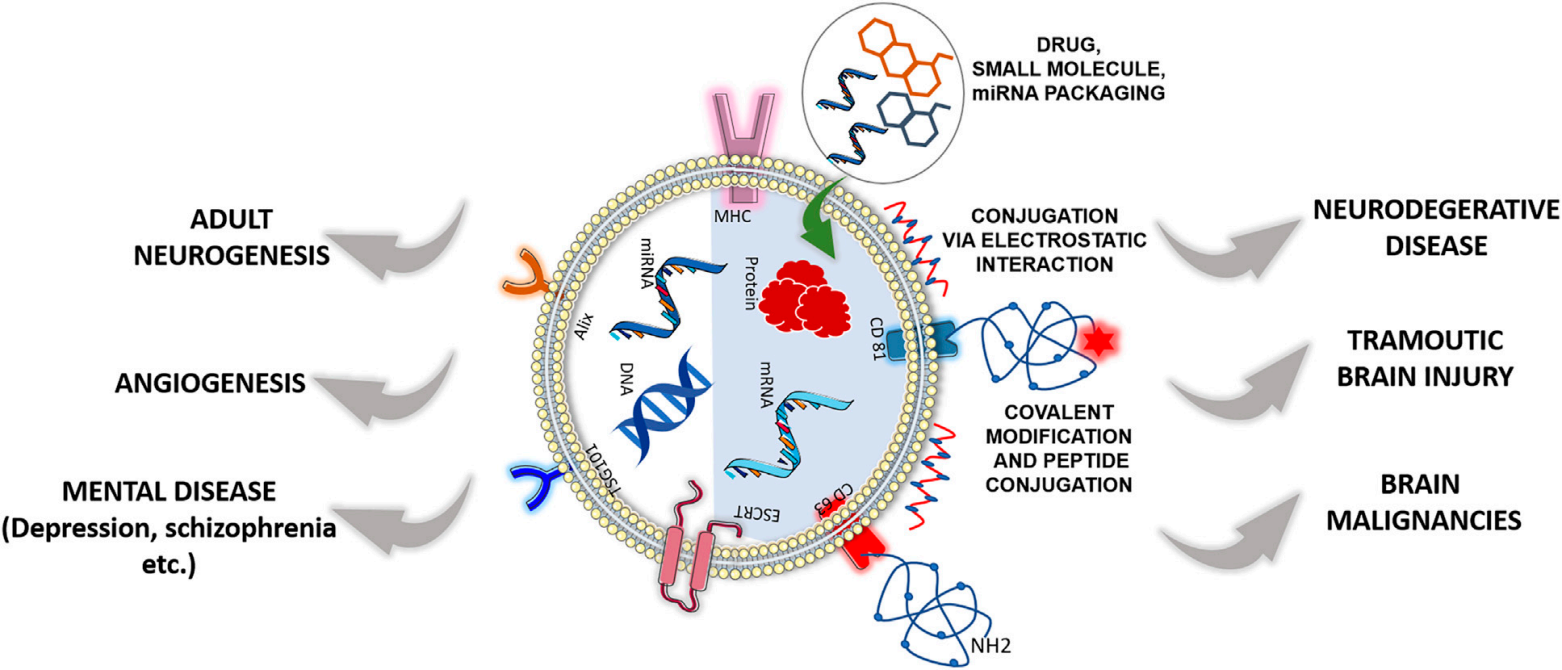
Therapeutic effect of designer exosomes or cell-derived natural exosomes in neurological disorders. The left half of the exosome represents cell-derived natural exosome, and the right half represents surface-functionalized engineered exosome. Both cell-derived and engineered exosomes have a therapeutic effect in neurological disorders with advantages and disadvantages. [Some component of the figure is adapted from Servier Medical Art; Servier is licensed under a creative commons attribution 3.0 unported license (https://smart.servier.com/)].

Natural exosomes have various potentials; their clinical application is associated with some inherent limitations of targetability, immunogenicity, and less efficient cargo delivery. Recently, to overcome these limitations, exosome engineering and the development of designer exosomes are coming into the picture. In this section, we will first discuss the role of natural exosomes as neurotherapeutics, and toward the end, we will discuss the role of surface engineered novel cargo-loaded exosomes as neurotherapeutics (Figure 5). Mesenchymal stem cell (MSC) is already extensively studied for regenerative medicine, cell therapy, and tissue engineering (Ankrum and Karp, 2010). Accordingly, research based on exosomes derived from MSCs (MSC-exosomes) has great value as this has the advantage of exosomes and also the characteristics of MSCs (Ghosh et al., 2020). Numerous studies have demonstrated the therapeutic value of MSC-exosomes in tumors, neurodegenerative diseases, cardiovascular and cerebrovascular diseases, wound repair, etc (Cui et al., 2018; Zhao et al., 2018; Moon et al., 2019; Rao et al., 2019; Riazifar et al., 2019). MSC-derived exosomes can exert their therapeutic effect by removing or inhibiting pathological processes or by promoting regenerative mechanisms. In the former case, it is known to reduce amyloid-beta (Aβ) aggregate in AD, rescue dopaminergic neurons from 6-OHDA-induced apoptosis in PD, reduce demyelination in multiple sclerosis (MS), and inhibit apoptosis, inflammation, and promotes angiogenesis in TBI and stroke (Jarmalavičiūtė et al., 2015; Huang et al., 2017; Yang et al., 2017b; Ding et al., 2018; Elia et al., 2019). In the latter case of regeneration, MSC-derived exosomes exert their effect by neuroprotection, neurogenesis, neuromodulation, and angiogenesis in many disease conditions like AD, TBI, and stroke (Doeppner et al., 2015; Zhang et al., 2015; Elia et al., 2019; Otero-Ortega et al., 2020). Other than the aforementioned process, MSC-exosomes show their effect in reducing oxidative stress, restoring the integrity of the BBB, inhibiting tumor growth, and improving behavioral and biochemical deficits in mental disorders like schizophrenia (Jarmalavičiūtė et al., 2015; Chen et al., 2016; Li et al., 2019; Tsivion-Visbord et al., 2020; Luo et al., 2021). Apart from MSC-exosomes, other cell-derived exosomes are explored for a similar application. Sharma et al. (2019) have shown exosomes released by neural cultures can rescue deficits in neuronal proliferation, differentiation, synaptogenesis, and synchronized firing in MECP2-knockdown human primary neural cultures, a model for Rett syndrome. Chen et al. (2020b) have shown exosomes derived from astrocytes promoted the recovery of damaged neurons by downregulation of the apoptosis rate and upregulating mitochondrial function. Lopez-Verrilli et al. (2013) have shown Schwann cells (SC)-derived exosomes increase axonal regeneration *in-vitro* and increase regeneration after sciatic nerve injury *in vivo*. Webb et al. (2018) demonstrated that human neural stem cell-derived EVs improve behavior and mobility by removing intracranial hemorrhage, reducing the volume of the cerebral lesions and brain swelling, which eventually leads to recovery from ischemic stroke in a pig model. From the previous discussion, it is clear that natural exosomes have a potential therapeutic effect; many attempts have been made to improve the therapeutic potential and load cargo of interest. Alvarez-Erviti et al. (2011b) have generated engineered dendritic cells that express Lamp2b fused to the neuron-specific RVG peptide and isolated exosomes from the engineered cell. They have packed the exosomes with exogenous siRNA by electroporation and demonstrated the targeted delivery of cargo specifically to neurons, microglia, and oligodendrocytes in the brain, resulting in a specific gene knockdown and subsequent therapeutic effect in AD (Alvarez-Erviti et al., 2011b). Using a similar approach, Yang et al. (2017a) have successfully delivered miR-124 to the infarct site, which leads to amplification of adult neurogenesis in ischemia. Tian et al. (2018) have developed c (RGDyK)-conjugated curcumin-loaded exosomes (cRGD-Exo), which can target the lesion region and show strong suppression of the inflammatory response and cellular apoptosis in the lesion region of the ischemic brain after intravenous administration. A group of scientists developed superparamagnetic iron oxide nanoparticles (SPIONs) and curcumin (Cur)-loaded exosomes. By click chemistry, they have conjugated the exosome membrane with neuropilin-1-targeted peptide (RGERPPR, RGE); these engineered exosomes have the ability to target glioma cells and also have imaging and therapeutic functions (Jia et al., 2018). Ye et al. (2018) have developed methotrexate (MTX)-loaded EVs functionalized with therapeutic [Lys-Leu-Ala (KLA)] and targeted [low-density lipoprotein (LDL)] peptides which show targetability toward brain tumors and show therapeutic effects. Wang and Han (2019) have modified the exosomes and loaded a plasmid expressing B-cell lymphoma-2 (Bcl-2) and Bcl-2-associated X-protein (Bax) short hairpin RNA (shRNA); these exosomes show therapeutic effect in apoptosis and neural functions after TBI. Zhang et al. (2019) have developed c (RGDyK) peptide conjugated, cholesterol-modified miR-210 engineered exosomes, which show upregulated expressions of integrin β3, vascular endothelial growth factor (VEGF), and CD34 and subsequent angiogenesis after middle cerebral artery occlusion (MCAO). In addition to different scientific groups, many companies are developing exosome-based neurotherapeutics; one such company is Evox Therapeutics. Evox Therapeutics uses a biotechnological-based approach to generate drug-loaded brain and central nervous system targeted engineered exosomes (https://www.evoxtherapeutics.com/). Aruna Bio is working on pharmaceutical exosomes for drug delivery to the brain and neurons (https://www.arunabio.com/). Some major disadvantages of cell therapy (induced pluripotent stem cell, iPSC), such as necrosis or abnormal cell differentiation, tumorigenesis, immune rejection caused by cell transplantation, and microvascular embolism, can be overcome by exosome-based therapies (Ghosh et al., 2020). The main advantages of exosome therapies are as followed. First, exosomes mediate stem cell paracrine action, participate in cell–cell communication and are already proven as the main mechanism of disease treatment mediated by cell-based therapies. Second, exosomes can be engineered and can be packed with a cargo of interest like existing, newly developed compositions, and can work as drug delivery vehicles. Third, in some cases, exosomes have autonomous targeting capabilities which make exosome-specific tissue or cell-targeted drug carriers (Mathieu et al., 2019; Wei et al., 2021). From the aforementioned studies, we can have an idea that exosome-based neurotherapeutics have made huge progress in recent years and with the development of new technologies, more progress will follow in upcoming days and can be an alternative to cell-based therapies, like iPSC therapies.

## 5 Discussion and Future Perspective

Exosomes are a rising star and a complete package in the era of advanced medical science due to their multiple roles in cell–cell communication, biomarker discovery, disease progression, and therapeutics. The following are some of the benefits that exosomes have: 1) they can pass the blood–brain barrier, and are less invasive, non-tumorigenic, and non-immunogenic, 2) their shelf life and half-life in patients are longer, allowing for long-term storage without loss of function, and 3) they do not reproduce or cause a microvascular embolism (Ghosh et al., 2020). These advantages make the exosomes a superior tool for biomarker discovery and therapeutic development. Apart from advantages, the main challenges for bringing exosomes into the clinical practice include the following: First, the urgent need for standard, efficient, and sensitive methods with a low biofluid volume requirement and high purity and yield for classification and extraction of exosomes from different body fluids and cells. Second, the identification of specific subtypes of EVs is urgently needed, as different vesicles may exert various biological effects. Current methods of exosome isolation are too diverse to confirm the purity of the product. Therefore, it is necessary to standardize the protocols and identification methods when attempting to use exosomes widely in clinical testing. Additionally, more reliable biomarkers should be confirmed. Although many molecules carried by exosomes have been documented to serve as potential biomarkers, few of them are qualified for application. Documented biomarkers need to be validated on a larger scale to create a standard hallmark for diseases. Third, for exosome-based therapeutics development, the targetability of exosomes needs to be checked, as different culture conditions change exosomal cargo. A standardized protocol needs to be developed for large production of exosomes from the cell; cell banks need to be developed. A specific purification method and sensitive method for specific exosomal marker identification need to be developed to avoid ambiguity. Last but not least, the biological safety, targeted efficacy, and adverse effects of exosomes must be confirmed before clinical use. In recent years, to endow the exosomes with targetability and to make exosomes more potent delivery and therapeutic agents, exosome engineering is coming into the picture, which will resolve many issues that cell-derived exosomes have. In conclusion, if the abovementioned lags are resolved and guidelines prescribed by the International Society for Extracellular Vesicles (Théry et al., 2018) are followed, then exosomes can be in the spotlight of clinical practice as biomarkers and therapeutics in the near future.
