# Supplementary material for: Exosome: The “Off-the-Shelf” Cellular Nanocomponent as a Potential Pathogenic Agent, a Disease Biomarker, and Neurotherapeutics
Source: Front Pharmacol. 2022 May 24;13:878058. doi: 10.3389/fphar.2022.878058 (PMC9170956; doi:10.3389/fphar.2022.878058)
Supplement: Supplementary file 1 [file Table1.docx]

| Exosome extraction method | Quantity of Biological fluid | Advantages/  Disadvantages | Quality controls | | Experiment | References |
| --- | --- | --- | --- | --- | --- | --- |
| **Ultracentrifugation**:  Particles with different density and size will show different sedimentation rate under centrifugal force. | Plasma: ~ 4ml; Serum: ~200 μl; CSF: ~2-5 ml | **Advantage:**  Low cost and Low contamination risk with unwanted reagents; suitable for large volume preparation.  **Particle concentration, and size**:  Plasma: (2.42 ± 2.44) × 10^9^ particles/ml size 121.5 ± 3.2 nm, recovery rate 22.72 ± 2.54 %.  Serum:6.27 × 10^9^ ± 4.66 × 10^8^ particle/ml size 92.9 ± 2.4 nm.  CSF: 1.06×10^9^±1.18×10^8^ particles/ml, size 137.83±5.95 nm.  **Disadvantage:**  Potential mechanical damage due to high-speed centrifugation, not suitable for small volume diagnosis, Time consuming, Labour intensive. | | Sequential centrifugation is important to remove unwanted particle and filtering with 0.22-micron filter may increase purity of isolated exosome. | RNA and proteomics profiling for biomarker discovery. | (Brennan et al., 2020;Dong et al., 2020;Li et al., 2021) |
| **Density gradient ultracentrifugation**:  After centrifugation in a dense medium, particle will stay in the position of the medium where it will get similar density. | Plasma: 0.5-3ml; Serum: ~700 μl; CSF: 5–8 mL | **Advantage:**  High purity, separation of subpopulation of exosomes is possible.  **Particle concentration, and size:**  Plasma: 10^10^ particles/ml  Size ~30- 100 nm  Serum: 4.93 × 10^9^ ± 2.07 × 10^9^ particles /ml, size 130.7 ± 5.9 nm  CSF: **~** 5 x 10^9^ particles / ml 20-100 nm  **Disadvantage:**  Low volume can be processed, time consuming method, mechanical damage due to high-speed centrifugation, large volume of sample required. | | Combination of cushion ultracentrifugation (CUC) or size exclusion chromatography with Density gradient ultracentrifugation can give better result. | Biomarker study, cellular uptake study for drug delivery. | (Street et al., 2012;Brennan et al., 2020;Wang et al., 2020;Holcar et al., 2021) |
| **Ultrafiltration**:  This method utilizes filter membrane with defined size-exclusion limit or molecular weight cut-off to separate mixture of particle. | Plasma:0.5 ml  Serum: ~200 μl,CSF:~ 5 ml | **Advantage:**  Lower equipment cost, Fast method, portability  **Particle concentration, and size:**  Plasma:  1.05 ± 0.50 × 10^11^ particles / ml, size 128.5 ± 3.1 nm.  Serum: (--)  CSF: (--)  **Disadvantage:**  Loss of sample due to clogging and membrane trapping, deformation of vesicles | | pre-treatment with proteinase can reuse fluid viscosity and tangential flow filtration can prevent sample loss. | Exosome based Therapeutics development and cellular uptake-based assay, biomarker discovery | (Dong et al., 2020) |
| **Precipitation**:  Change in the solubility of exosomes by hydrophilic water-excluding polymers. | Plasma: 0.5 ml; Serum: ~ 1 mL; CSF: 5 ml | **Advantage:**  Minimal hands-on time, Rapid, Specialised equipment not necessary, high yield of exosomal RNA  **Particle concentration, and size:**  Plasma:(2.70 ± 2.10) × 10^11^ Particles /ml, size 107.5 ± 2.6 nm, recovery rate 56.44 ± 12.12%.  Serum: 1.66 x 10 ^9^ -5.17 x 10^9^ particles / ml, size renges from 98.7 ± 1.9 nm - 104.7 ± 4.0 nm.  CSF: ~6.3 x 10^7^ particles/ml, size ranging from 126.1±40.0 nm - 155.0±19.8 nm  **Disadvantage:**  Contaminants of protein aggregates, other extracellular vesicles and polymeric contaminants, Require complicated clean-up steps. | | pre-cleaning of samples is required to remove cells and cellular debris. Incubation time and temperature is critical factor. | Exosomal miRNA profiling | (Rekker et al., 2014;Soares Martins et al., 2018;Dong et al., 2020;Neerukonda et al., 2020) |
| **Size-exclusion chromatography**:  Elution of particle through porous materials in accordance with their particle size, big particles eluted earlier and small will elute latter. | Plasma:~ 0.5 ml;Serum: ~200 μl; CSF: ~5 ml | **Advantage:**  High purity, Fast preparation, no morphological change in exosomes, reproducibility, applicable for both small and large sample volume.  **Particle concentration, and size**:  Plasma: 1.4 x 10^11^- 6.5 x 10^11^ particles /ml, size ~ 20-100nm  Serum: --  CSF: --  **Disadvantage:**  Need to be used in combination with other isolation method to get expected result. | | Better result when combination of Size-exclusion chromatography and ultrafiltration is used. | Coding, Noncoding RNA and protein profiling. | (Welton et al., 2017;Hermann et al., 2019;Wei et al., 2020;Holcar et al., 2021) |
| **Microfluidic**:  Based on different principles including immunoaffinity, size, density, tangential flow filtration etc. | Plasma: ~ 500 μl;  Serum: 10–400 μl;  CSF: ~2-5 ml | **Advantage**:  high recovery rate, high purity (> 97 %), fast, absence of any reagents that can affect biological activity of exosomes, easily automated & integrated with diagnosis.  **Particle concentration, and size**:  plasma:(1.08 ± 0.95) ×10^11^ particles/ ml, size 138.1 ± 35.23 nm and recovery rate of > 80%.  Serum:4.83 × 10^10^ ± 3.85 × 10^9^ particles/ ml size 20-140 nm, recovery rate of 42- 94%.  CSF: 4.74×10^9^±3.56×10^8^ particles/ ml, size 140.60±6.96 nm.  **Disadvantage**: Device cost, Low sample capacity | | Combining centrifugation and microfluidics to get better purity | Proteomics, DNA, RNA, Noncoding RNA analysis for biomarker discovery. Cellular uptake assay for drug delivery perpose. | (Chen et al., 2010;Hisey et al., 2018;Han et al., 2021;Li et al., 2021) |
| **Immuno capture**:  Based on specific binding between exosome markers and immobilized antibodies/ ligand. | Plasma: ~1 mL;Serum: ~200 μl; CSF: ~ 5 ml | **Advantage:**  Suitable for separating exosomes of specific origin; High-purity exosomes Easy to use  No chemical contamination  **Particle concentration, and size:**  Plasma: 1.1 ± 0.3 x 10 ^11^  Particles/ml, purity 96 %, size ~ 100 nm  Serum: 1 x 10^10^ particle / ml, size ~ 100 nm  CSF: --  **Disadvantage:**  Lack of specific markers for isolation, Difficulty separating EVs from antibodies or beads | | Choosing appropriate antibody to purify exosome of interest. | miRNA profiling and biomarker discovery to predict disease sates. | (Yoo et al., 2012;Shtam et al., 2020) |

Supplementary Table 1: Tabular representation of different exosome isolation method from different biofluids. The table showcases principle, advantages, disadvantages, yield, quality control and types of experiment can be done from the isolated exosome using different method. (“--” denotes no related article found.)
