# Supplementary material for: Exosome: The “Off-the-Shelf” Cellular Nanocomponent as a Potential Pathogenic Agent, a Disease Biomarker, and Neurotherapeutics
Source: Front Pharmacol. 2022 May 24;13:878058. doi: 10.3389/fphar.2022.878058 (PMC9170956; doi:10.3389/fphar.2022.878058)
Supplement: Supplementary file 2 [file Table2.docx]

| **Biofluid: Disease state** | **Exosome Content/Biomarker and Remark** | **Reference** |
| --- | --- | --- |
| Blood plasma: AD | Presence of elevated level of tau, P-T181-tau, P-S396-tau, and amyloid β 1-42 (Aβ1-42) in brain derived blood exosomes can predict AD 10 years before clinical onset.  Upregulation of cathepsin D, lysosome-associated membrane protein 1 (LAMP-1), and ubiquitinylated protein and downregulation of heat-shock protein 70. This denotes neuronal lysosomal dysfunction is an early event in the development of AD  Elevated level of β-site amyloid precursor protein-cleaving enzyme 1 (BACE-1), γ-secretase, soluble Aβ42, soluble amyloid precursor protein (sAPP)β, sAPPα, glial-derived neurotrophic factor (GDNF), P-T181-tau, and P-S396-tau in astrocyte-derived exosomes (ADEs)  Lower miR‑193b level.  Decreased level of miR-212 and miR-132  Growth associated protein 43 (GAP43), neurogranin, synaptotagmins, Rab3A, and synaptosome associated protein 25 | (Fiandaca et al., 2015;Jia et al., 2019) (Winston et al., 2016)  (Goetzl et al., 2015)  (Goetzl et al., 2016)  (Liu et al., 2014)  (Cha et al., 2019)  (Jia et al., 2021) |
| Blood plasma: PD | Elevated level of α-synuclein in plasma exosomes as compared to CSF exosomes.  higher level of L1CAM exosomal tau.  Increased level of lncRNA POU3F3 and α-syn in plasma L1CAM exosomes  Increased DJ-1 and α-synuclein | (Shi et al., 2014;Wang et al., 2018)  (Shi et al., 2016)  (Zou et al., 2020)  (Zhao et al., 2019) |
| Blood plasma: ALS | TAR DNA-binding protein-43(TDP-43)  Upregulation of miR-146a-5p, miR-151a-5p, miR-199a-3p, miR-199a-5p, miR-151a-3p and downregulation of miR-29b-3p, miR-10b-5p, miR-4454 | (Chen et al., 2020)  (Banack et al., 2020) |
| Blood plasma: FTD | Elevated level of P-T181-tau and Aβ1-42  Higher HSP70 levels | (Fiandaca et al., 2015)  (Chanteloup et al., 2019) |
| Blood plasma: Mental Disorder | Decrease in level of miR-484, miR-652-3p, and miR-142-3p and increased level of miR-185-5p in bipolar disorder (BD).  Elevated level of miR-206 in Schizophrenia (SCZ). | (Ceylan et al., 2020)  (Du et al., 2019) |
| Blood plasma: TBI | Elevated level of Aβ42 and lower level of neurogranin (NRGN) in NDE and ADE.  NfL level | (Winston et al., 2019)  (Guedes et al., 2020) |
| Blood plasma: GBM | Decreased level of IFN-γ, IL-10, and IL-13  EGFRvII, EGFR, podoplanin (PDPN) | (Cumba Garcia et al., 2019)  (Shao et al., 2012) |
| Serum: AD | Upregulation of miR-135a and miR-384 and downregulation of miR-193b.  miR-30b-5p, miR-22-3p, and miR-378a-3p | (Yang et al., 2018)  (Dong et al., 2021) |
| Serum: PD | Downregulation of miR-19b upregulation of miR-195 and miR-24 | (Cao et al., 2017) |
| Serum: ALS | Downregulation of miR-27a-3p | (Xu et al., 2018) |
| Serum: FTD | Upregulation of miR-106b-5p, miR-20a-5p, mir-106b~25, miR-30d-5p and mir-17 | (Cheng et al., 2015;Denk et al., 2018) |
| Serum: Mental Disorder | Decreased exosomal brain derived neurotrophic factor (BDNF) levels and increased proBDNF levels in major depression (MD).  Increased insulin receptor substrate −1 (IRS-1) in L1CAM+ exosomes in major depressive disorder (MDD). | (Gelle et al., 2021)  (Nasca et al., 2020) |
| Serum: TBI | Higher level of NFL and GFAP in patients with diffuse injury than in patient with focal lesions.  UCH-L1 involves in early mortality. | (Mondello et al., 2020) |
| Serum: GBM | Polymerase I and transcript release factor (PTRF)  miR-320, miR-574-3p, miR-301a, miR-151a, miR-148a, miR-21  Upregulation of miR-21, miR-222 and miR-124 3p | (Huang et al., 2018)  (Jiang et al., 2018)  (Santangelo et al., 2018) |
| CSF: AD | HSPA1A, NPEPPS and PTGFRN for progression of mild cognitive impairment (MCI) to AD.  Lower miR‑193b level.  miR-125b-5p, miR-451a, and miR-605-5p | (Muraoka et al., 2020)  (Liu et al., 2014)  (McKeever et al., 2018) |
| CSF: PD | α-synuclein oligomer in CD11b+  exosome pS1292-LRRK2 | (Guo et al., 2020)  (Wang et al., 2017) |
| CSF: ALS | CUE domain-containing protein-2 (CUEDC2)  TDP-43  High level of novel INHAT repressor (NIR) | (Otake et al., 2019)  (Feneberg et al., 2014)  (Hayashi et al., 2020) |
| CSF: FTD | Lower level of miR-320a, miR-328-3p, and miR-204-5p. | (Tan et al., 2021) |
| CSF: Mental Disorder | -- | -- |
| CSF: TBI | Total tau (t-tau) and tau phosphorylated on threonine181 (p-tau181) | (Muraoka et al., 2019) |
| CSF: GBM | Elevated level of miR-21 | (Akers et al., 2013) |

Supplementary Table 2: Tabular representation of exosomal biomarker in neurological diseases (AD: Alzheimer’s disease; PD: Parkinson Disease; ALS: Amyotrophic Lateral Sclerosis; FTD: Frontotemporal Dementia; TBI: Traumatic brain injury; GBM: Glioblastoma multiform; CSF: Cerebrospinal fluid.)(“ -- ” denotes no related article found.)
